# Development of a target product profile for new glucose self-monitoring technologies for use in low- and middle-income countries

**DOI:** 10.1371/journal.pone.0309062

**Published:** 2024-08-26

**Authors:** Elvis Safary, Molly Lepeska, David Beran, Margaret Ewen, Aigerim Zhaparova, Jackie Rukare, Philippa Boulle, Sigiriya Aebischer Perone, Solomzi Makohliso, Stefan Pleus, Beatrice Vetter

**Affiliations:** 1 FIND, Geneva, Switzerland; 2 Health Action International, Amsterdam, Netherlands; 3 Division of Tropical and Humanitarian Medicine, University of Geneva and Geneva University Hospitals, Geneva, Switzerland; 4 Independent Consultant, Bishkek, Kyrgyzstan; 5 Independent Consultant, Kampala, Uganda; 6 Médecins Sans Frontiéres, Geneva, Switzerland; 7 International Committee of the Red Cross, Geneva, Switzerland; 8 Essential Tech Centre, École Polytechnique Fédérale de Lausanne, Lausanne, Switzerland; 9 Institut für Diabetes Technologie Forschungs- und Entwicklungsgesellschaft mbH an der Universität Ulm, Ulm, Germany; Stellenbosch University, SOUTH AFRICA

## Abstract

**Aims:**

Most glucose self-monitoring devices have been developed with high-income countries in mind. We developed a target product profile (TPP) for new glucose self-monitoring technologies for users in low- and middle-income countries (LMICs).

**Methods:**

A draft TPP including 39 characteristics was developed by an expert group including diabetes specialists, device specialists, and people with diabetes, incorporating findings from qualitative research in LMICs. Each characteristic had minimal and optimal requirements for two use cases, frequent and sporadic use. Characteristics requiring refinement were identified via online survey. Characteristics with agreement level <90% for any requirement were reviewed by the expert group and amended as appropriate.

**Results:**

One characteristic (shelf life) had agreement <75% (both requirements for both use cases). Characteristics with agreement ≥75% and <90% for the frequent use case included infrastructure level, measurement cycle, duration of use before replacement, interchangeability, and calibration (both requirements), and activity log and price per month to end payer (minimal requirement). Intended use (both requirements), accuracy, and price per month to end payer (optimal requirement) had agreement ≥75% and <90% for the sporadic use case.

**Conclusions:**

This TPP will inform developers on requirements for glucose self-monitoring technologies for LMICs, and support decision-makers in evaluating existing devices.

## Introduction

Of approximately 537 million people worldwide living with diabetes in 2021, the majority were living in low-and middle-income countries (LMICs) [1]. The prevalence of diabetes has been increasing steadily over the past few decades and is predicted to reach over 783 million by 2045 [1, 2]. Strengthening the management of diabetes in LMICs is imperative to prevent serious complications, such as cardiovascular disease, blindness, kidney disease, nerve damage, and lower limb amputation, as well as early death [1].

Self-monitoring of blood glucose, i.e. the regular collection of detailed information on blood glucose levels at numerous timepoints throughout the day, is considered an integral part of self-management for people living with diabetes, especially for people with type 1 diabetes, and people with type 2 diabetes either on insulin therapy, or using non-insulin glucose-lowering drugs that can induce hypoglycaemia [3]. Conventional self-monitoring of blood glucose requires a fingerprick blood sample, which is analysed using a blood glucose meter. As this can be associated with pain and inconvenience, there is more demand for devices that enable glucose monitoring without blood sampling, such as optical monitors, subcutaneous sensors, or devices that use other sample types [4]. Such devices may enable more frequent sampling, leading to greater data availability. Most development of medical devices takes place in high-income countries (HICs), with the specific attributes of high-income users and markets guiding product characteristics [5, 6]. The suitability of devices for use in LMICs is often overlooked [6], and access to appropriate devices in these settings is limited. Thus, there is a need for guidance for developers on the desirable attributes of glucose self-monitoring devices for use in LMICs.

A target product profile (TPP) defines the minimal and optimal requirements for a device. TPPs have frequently been used to guide developers and manufacturers on the development of diagnostics for infectious diseases [7–12], and more recently for digital tools, applications and multi-parameter diagnostic devices [7–17]. Here, we describe the development of a TPP for new glucose self-monitoring technologies for management of diabetes in LMICs. This included technologies that measure glucose continuously through a minimally invasive procedure, optical monitors, subcutaneous sensors, or devices that use other sample types. The aim was to provide a TPP that takes into consideration the specific needs, preferences and resources of people living in LMICs, including environmental conditions, infrastructure, and access to healthcare. Devices developed in line with such a TPP could improve the ability of people living with diabetes in LMICs to perform glucose testing according to their self-monitoring needs, potentially leading to improved glycaemic control and quality of life.

## Materials and methods

The TPP was developed in four stages: (1) preparation of a draft TPP by an expert group, (2) qualitative research in four LMICs (Kyrgyzstan, Mali, Peru and Tanzania) with people living with diabetes, their caregivers and healthcare providers, (3) consensus building through an online survey to identify device characteristics for further refinement, and (4) TPP finalization by the expert group. The TPP was limited to non-invasive or minimally invasive devices [4]. This included technologies that measure glucose continuously through a minimally invasive procedure (i.e. one time insertion of a sensor into the subcutaneous tissue), a non-invasive optical or other spectroscopic procedures (e.g. measurement via optical means through the skin), or a non-invasive fluid-sampling procedure (e.g. sweat sampling via a patch). It also included non-invasive technologies that do not measure glucose levels continuously, e.g. application of a strip to the tongue for glucose measurement in saliva or temporary application of an optical device to the body. The TPP excluded any technology that requires implantation or direct sampling of blood for measurement.

As the TPP development did not include human or animal subjects, no ethical committee approval, review board approval, or informed consent was required. Ethical approval was obtained for the qualitative research conducted in Kyrgyzstan, Mali, Peru and Tanzania. Results of this research are reported in a separate publication [18]. There are no specific standards or guidelines for the development of TPPs for diagnostics, however, the methodology used in this study was consistent with protocols for previous TPPs developed by FIND and partners [7–17].

### Draft TPP development and qualitative research

A group of 13 experts was convened to develop the draft TPP. This group was comprised of experts from FIND, Health Action International (HAI), the Swiss Federal Institute of Technology Lausanne (EPFL), the International Committee of the Red Cross (ICRC), the Institut für Diabetes-Technologie (IfDT), Medicines Sans Frontiers (MSF), the University of Geneva and Geneva University Hospitals (UNIGE), the World Health Organization (WHO), and people living with diabetes (Table 1).

**Table 1:** Characteristics of the expert group.

Organization	Relevant expertise/role	Qualifications
FIND	NCD director	PhD
FIND	NCD scientist	PhD
EPFL	Chief Strategic Officer, biomedical engineer and medical sciences	PhD
HAI	Expert in medicine pricing, pharmacist	PhD
HAI	Program manager, communication expert, person living with type 1 diabetes	MSc
ICRC	Specialist in general internal medicine, and humanitarian conflict settings	MD
IfDT	Head of scientific operations	PhD
Independent	Consultant, person living with type 1 diabetes	MPP
Independent	Marketing and business consultant and CEO, diabetes educator and advocate, parent of person living with type 1 diabetes	MBA
MSF	NCD advisor and working group lead, humanitarian conflict physician	MPH
UNIGE	Lecturer and researcher, public health specialist	MD, PhD
WHO	Team lead for medical devices and IVDs, biomedical engineer	MSc
WHO	Medical officer	MD, PhD

CEO, Chief Executive Officer; IVDs, *in vitro* diagnostics.

The scope for the TPP was defined at a virtual workshop that took place on 6 May 2021. A second virtual workshop took place on 11 June 2021 to review the draft TPP requirements. An initial draft (version 0) of the TPP was developed as a result of these two meetings. Version 0 included two use cases, frequent users and sporadic users. Frequent users were defined as people living with diabetes who were on insulin, and who measure glucose at least two times per day, every day (as well as individuals with diabetes who frequently test during a limited number of weeks/months during special circumstances, such as pregnancy). Sporadic users were defined as people living with diabetes who were not on insulin and who measure glucose once per day or less. Each use case had 42 characteristics, and each characteristic had minimal and optimal requirements. The characteristics were divided into six higher-order categories: scope, device, utility requirements, performance and results, purchasing considerations, and safety and standards.

Version 0 was used as a baseline to conduct a qualitative research study in Mali, Peru, Tanzania, and Kyrgyzstan, aimed at collecting input from people living with diabetes, their caregivers and healthcare providers. This was a mixed-methods, exploratory, qualitative study, using individual in-depth interviews, focus group discussions and participatory action research to for structured data collection on the TPP characteristics and requirements. A total of 383 people participated in the study and results are reported in a separate manuscript [18]. The study took place between February and July 2022.

A third workshop took place in-person in Geneva, Switzerland, on 28–29 September 2021. The aim of this workshop was to refine the version 0 TPP requirements based on the findings of a qualitative research study. As a result, characteristics that were closely related were merged to improve clarity and to allow easier feedback during the survey, leading to a reduction in number of characteristics to 39 resulting in a revised version of the TPP (version 0.1).

### Consensus building

Following the three initial workshops, a two-step Delphi-like process was employed to facilitate consensus building for the TPP. Firstly, the draft TPP was reviewed through an online survey. Secondly, the survey results were reviewed by the expert group and amended as appropriate.

The online survey was open to anyone with access to the internet. The first round of the survey was made available through the Alchemer survey tool in four different languages (English, Spanish, French and Russian) from 3 March to 16 April 2023. Links to the survey were posted by FIND on all social media channels (LinkedIn ~30,000 followers, Facebook >7,000 followers and Twitter >13,000 followers). The link was also shared via email to the FIND general mailing list and to partners who posted the link via their social media platforms. Members of the expert group also distributed the link amongst their respective networks. The targeted audience included people living with diabetes, diabetes advocates, device experts, clinicians and nurses, public health agencies, non-governmental organization personnel and manufacturers. FIND also organized two optional short online webinars to support completion of the survey, which took place on the 8 March and 15 March 2023. There was no reward or incentive offered for completing the survey.

Survey respondents were asked to rate their level of agreement with each of the 39 minimum and 39 optimum requirements in the draft TPP (version 0.1) in their chosen use case and category using a 4-point Likert scale (1 = fully disagree, 2 = mostly disagree, 3 = mostly agree, 4 = fully agree), including an option to answer `no opinion’. Respondents were free to choose the categories and use cases that they wanted to answer. The percentage agreement with each requirement was determined by the number of respondents with a ‘mostly agree’ or ‘fully agree’ rating. Disagreement with a criterion was based on a rating of ‘fully disagree’ or ‘mostly disagree’, and required a comment or suggestion from the survey respondent to explain their reasons for disagreement. Respondents could provide additional comments to accompany scores of ‘mostly agree’ or ‘fully agree’ if desired, but this was not mandatory.

Following the first round of the survey, a fourth expert group virtual workshop took place on 17 May 2023. The objective was to review characteristics/requirements with a pre-specified consensus threshold of <75% agreement, corresponding to a Likert score of either 1 or 2 for ≥75% of respondents. However, only one characteristic had an agreement level of <75%, therefore, characteristics with agreement ≥75% and <90% were also reviewed. These requirements were adjusted based on respondents’ comments, as appropriate, resulting in a revised version of the TPP (version 0.2).

### TPP finalization

As the large majority of characteristics and requirements had a high level of agreement in the online survey, it was decided to forgo a planned second round of survey. Version 0.2 of the TPP was published for open consultation on the FIND website, and was also distributed using the same methods used for the online survey. The TPP was available for review from 16 June to 14 July 2023, following which any comments would be reviewed and incorporated, if appropriate, to create the final version of the TPP (version 1).

## Results

### Online survey

Of 75 people who accessed the online survey, 40 people responded (20 fully completed and 20 partially completed). Respondents were from 18 countries. Of 28 respondents from LMICs, 13 fully completed and 15 partially completed the survey. In total, 18 respondents were people living with diabetes and 18 had more than 15 years’ experience in the field (Table 2).

**Table 2:** Characteristics of online survey respondents.

Characteristic	Number of respondents (N = 40)
**Country **	
Algeria	1
Bosnia & Herzegovina	1
Brazil	1
Congo	1
Ethiopia	1
France	2
India	7
Italy	1
Kyrgyzstan	3
Niger	1
Nigeria	1
Philippines	1
South Africa	6
Switzerland	2
Togo	1
Tunisia	1
Uganda	2
United States	4
No response	3
**Do you live with diabetes?**	
No	15
Yes, type 1	12
Yes, type 2 (on insulin)	2
Yes, type 2 (not on insulin)	4
Someone I care for lives with diabetes	6
No response	1
**Years of experience in the field**	
<1 year	1
1–4 years	6
5–9 years	6
10–14 years	6
>15 years	18
None	3
**Profession** ^**a**^	
Employee of NGO/associations	11
Industry	20
Medical doctor/diabetologist/endocrinologist	9
Government employee: public health agency	4
Advocate	18
Implementer	4
Biomedical engineer	2
Academia/Researcher	4

^a^Respondents could choose multiple answers. NGO, non-governmental organization.

**Fig 1:**
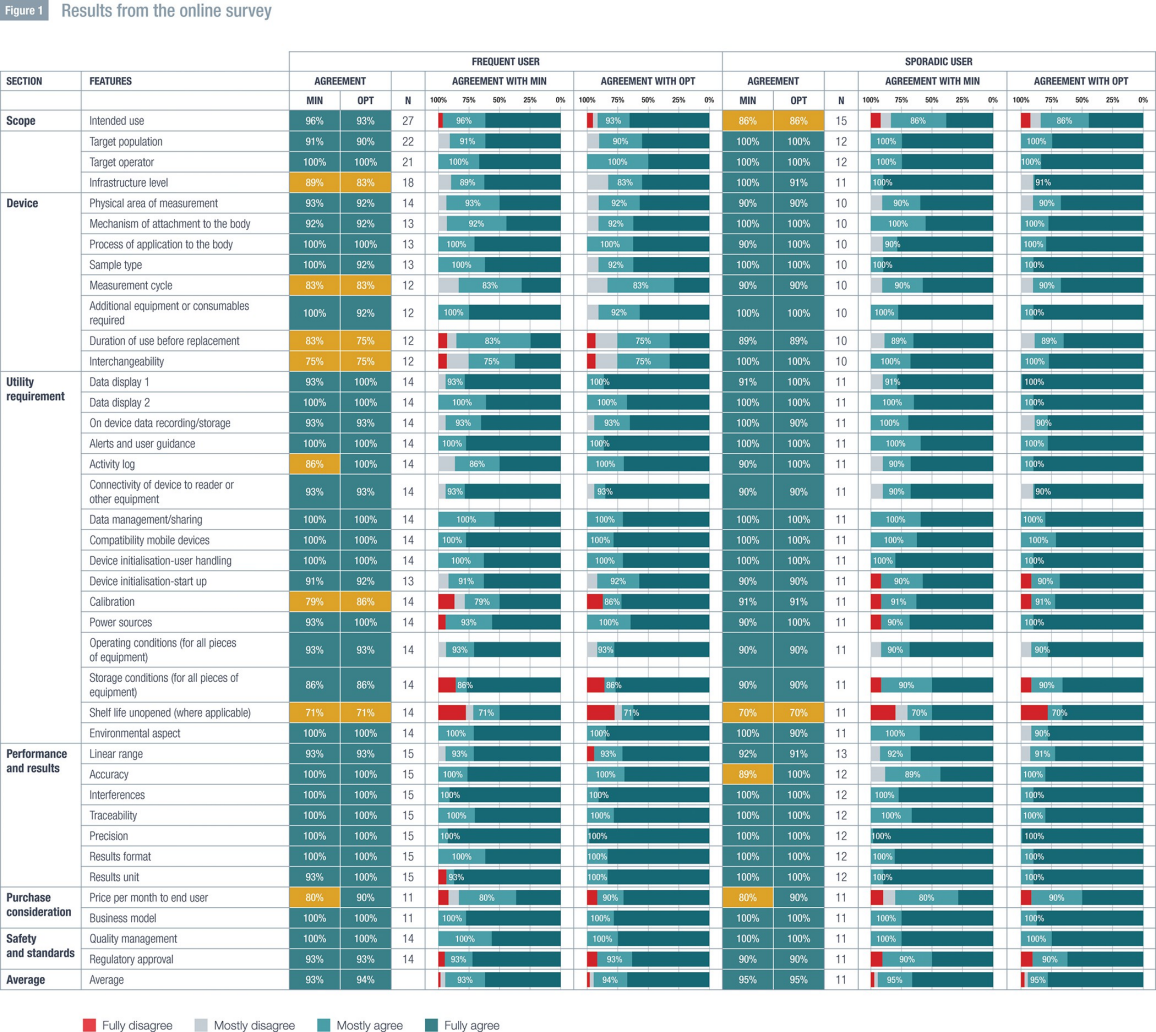
Results from the online survey. Min, minimal; opt, optimal.

The results from the online survey are shown in Fig 1. Of the 39 characteristics, only the shelf life characteristic had an agreement level <75%. Agreement for the shelf life characteristic was <75% for the minimal and the optimal requirements for both the frequent and sporadic use cases. Minimal characteristics with agreement level ≥75% and <90% for the frequent use case were infrastructure level (89%), activity log (86%), measurement cycle (83%), duration of use before replacement (83%), price per month to end user (80%), calibration (79%), and interchangeability (75%). Optimal characteristics with agreement level >75% and <90% for the frequent use case were calibration (86%), infrastructure level (83%), measurement cycle (83%), duration of use before replacement (75%), and interchangeability (75%). Minimal characteristics with agreement level >75% and <90% for the sporadic use case were accuracy (89%), intended use (86%), and price per month to end user (80%). The only optimal characteristic with agreement level >75% and <90% for the sporadic use case was intended use (86%).

### TPP refinement

There was disparity in the survey respondents’ comments on the shelf life characteristic. Some felt that the device should last as long as the technology has functional utility, while others thought that the minimal requirement of 24 months and optimal requirement of 36 months from date of production were too high. Following review, the expert group decided to maintain the shelf life at 24 and 36 months from date of production, as a shorter shelf life frequently results in products in country being close to the expiry date. For rapid diagnostic test TPPs, shelf life/stability requirements have ranged from 12 months for minimal to 18–24 months for optimal requirements [7, 9]. Appreciating that devices may have several components, additional specifications for multi-component systems (e.g. devices with a separate sensor and transmitter) were included for clarification, to state that the requirements apply to the component with the shortest shelf life.

Following review by the expert group, changes were also made to the following characteristics with agreement level >75% and <90%: interchangeability, calibration, duration of use before replacement, activity log and infrastructure level requirements (all frequent use case only), and price per month to end user (both use cases). The interchangeability characteristic was reworded to highlight user identification and functionality, stating that the device may be interchanged between users (e.g. sharing of an ear clip-based device), but that user identification functionality and storage of measurement and calibration data is required.

Survey respondents felt that user calibration was important to have as a minimal requirement, taking into account the fact that a degree of individualized calibration may be needed for certain devices. The term ‘factory calibrated’ was therefore removed from the requirement, and user calibration was included as desirable but not mandatory. For duration of use before replacement, respondents noted that for current devices, adhesives do not last well for longer than 2 weeks. A comment was therefore added to the requirement stating that adhesives need to be fit for the duration of use. For the activity log characteristic, survey respondents felt that the functions listed in the minimal requirement of version 0.1 may not be necessary for most users. As such, experts limited the minimal requirement to time-stamped symbols, with descriptive details of insulin injections, food intake, physical activity and other self-defined categories as the optimal requirement.

For the infrastructure level characteristic, survey respondents noted that battery powered devices may be most suitable for LMIC settings due to challenges in accessing a reliable power supply. Acknowledging that devices with batteries are available and may be developed in the future, the experts extended the wording on the minimal requirement to include a note on the need to use commonly available batteries, to minimize the risk of discontinued use due to inability to obtain suitable batteries. Power requirements for devices with disposable batteries should be minimal. The optimal requirement was changed to be the same as the minimal requirement, but with rechargeable batteries lasting an appropriate amount of time depending on device configuration.

While survey respondents commented that the minimal and optimal prices per month to end user in version 0.1 of the TPP (US$20 and $3.7, respectively, including one-off purchases of readers or other measurement devices) for both use cases were too high, the overall agreement rate with the suggested prices was 80% and higher (for both use cases, minimal and optimal). As such, the price was not adapted; however the wording was changed to ‘price per month to end payer’, to reflect the different individuals or entities that may be financially responsible for the cost of the product, which could be the end user, government, or insurer.

The measurement cycle and accuracy requirements for the frequent use case, and the intended use requirement for the sporadic use case, remained unchanged following expert review. Survey respondents commented that for measurement cycle, device manufacturers should set the frequency of measurement. However, during the qualitative study in LMICs, potential users expressed a desire for flexibility in measurement frequency. Therefore, the expert group decided to retain the minimal characteristic as user ability to determine measurement frequency, and the optimal as the same as the minimum, plus on-demand readings outside of the user-defined frequency. For intended use in the sporadic use case, respondents commented that semi-quantitative measurement of glucose would be acceptable. However, the expert group preferred to retain quantitative measurement in both the minimal and optimal characteristics, as the intended users are people not on insulin and those where self-monitoring is clinically indicated. In these situations, a discrete glucose value is more suitable for management of diabetes than a range of values.

### TPP finalization

No comments were received on version 0.2 of the TPP posted on the FIND website and this version was therefore considered to be final, resulting in TPP version 1 (**Table 3**).

**Table 3:** Finalized TPP for new glucose self-monitoring technologies.

Characteristics	Minimal	Optimal
**SCOPE**		
**Intended use**	**Frequent user**	
	Quantitative measurement of glucose in the product-specific sample type to be used for monitoring of glucose levels and adjustment of insulin; intended to replace fingerstick blood glucose testing	Same as minimum
	**Sporadic user**	
	Quantitative measurement of glucose in the product-specific sample type to be used for monitoring of glucose levels; intended to replace fingerstick blood glucose testing	Same as minimum
**Target population/ condition**	**Frequent user**	
	People with diabetes managed exclusively with insulin; should include the widest possible age range	Same as minimum and others with diabetes where short-term frequent monitoring might be indicated; there should be no age restriction
	**Sporadic user**	
	People with diabetes where self-monitoring is clinically indicated; should include the widest possible age range	Same as minimum and there should be no age restriction
**Target operator**	**Frequent user**	
	People with diabetes (adults and children) or their care providers able to react to results or alerts independently of their literacy or numeracy levels, visual capacity or cognitive ability	Same as minimum
	**Sporadic user**	
	People with diabetes (adults and children) or their care providers able to react to results or alerts independently of their literacy or numeracy levels, visual capacity or cognitive ability	Same as minimum
**Infrastructure level**	**Frequent user**	
	Can be used and store data without access to constant power supply, internet connectivity and water (for hygiene). If disposable batteries are used, the device power requirements should be minimal, and commonly available batteries should be used	Same as minimal with all batteries being rechargeable and charge lasting for an appropriate amount of time, considering device configuration.
	**Sporadic user**	
	Can be used and store data without access to constant power supply, internet connectivity and water (for hygiene). If disposable batteries are used, the device power requirements should be minimal, and commonly available batteries should be used	Same as minimal with all batteries being rechargeable and charge lasting for an appropriate amount of time, considering device configuration.
**DEVICE**		
**Physical area of measurement**	**Frequent user**	
	In an easily accessible area, not hindering daily activities	Flexible application to as many physical areas as possible for a specific device design, not hindering daily activates
	NOTES: No body part is specified to take into consideration different user preferences based on gender, sociocultural and environmental circumstances, which can differ widely	
	**Sporadic user**	
	In an area where the device can easily be applied to and removed from the body	Flexible application to as many physical areas as possible to an area where the device can easily be applied to and removed from the body
**Mechanism of attachment to the body**	**Frequent user**	
	For devices that are designed to be attached to the body: Held in place with a band, elastic or adhesive For devices that are not designed to be attached to the body: not applicable	For devices that are designed to be attached to the body: Same as minimum, including the option to remove the device temporarily For devices that are not designed to be attached to the body: not applicable
	**Sporadic user**	
	For devices that are designed to be attached to the body: Held in place with a band, elastic (excluding adhesives)	Same as minimum
**Process of application to body**	**Frequent user**	
	Can be autonomously attached by the user (without help by others); easily applied by users of any age or physical ability; providing immediate user-feedback for correct application	Same as minimum
	**Sporadic user**	
	Can be autonomously attached by the user (without help by others); easily applied by users of any age or physical ability; providing immediate user-feedback for correct application.	Same as minimum
**Sample type**	**Frequent user**	
	Any sample type that provides accurate results in any circumstance and is collected easily (sample types to consider are interstitial fluid, sweat, tears, saliva)	No sample material required
	**Sporadic user**	
	Any sample type that provides accurate results in any circumstance and is collected easily (sample types to consider are sweat, tears, saliva, urine)	Same as minimum
**Measurement cycle**	**Frequent user**	
	Ability of the user to determine frequency of measurement (including for devices designed to measure continuously)	Same as minimum, including on-demand reading outside of user-defined frequency.
	**Sporadic user**	
	Ability of the user to determine frequency of measurement	Same as minimum
**Additional equipment or consumables required**	**Frequent user**	
	No other equipment required than a device-specific reader or mobile phone to obtain readings	None, reading available on the device with optional transfer to reader, smartphone or cloud-based software.
	**Sporadic user**	
	None, reading available on the device with optional transfer to reader, smartphone or cloud-based software	Same as minimum
**Duration of use before replacement**	**Frequent user**	
	For devices using adhesives to attach to the body: 4 weeks. For all other devices (including readers): 3 years	For devices using adhesives to attach to the body: same as minimum. For all other devices (including readers): 5 years or longer.
	NOTES: Adhesives need to be fit for the duration of use	
	**Sporadic user**	
	For non-adhesive devices: 3 years	Same as minimum
**Interchangeability**	**Frequent user**	
	Can be interchanged between users (e.g. sharing of an ear clip-based device), requiring user ID functionality and storage of measurement and calibration data; user calibration possible put not required	Same as minimum
	**Sporadic user**	
	Unless minimally invasive or infection-control measures cannot be applied: Can be interchanged between users (e.g. sharing of an ear clip-based device) with minimum calibration; readers or accessories should be interchangeable between users; user ID functionality required, including storage of calibration and measurement data	Same as minimum
**UTILITY REQUIREMENT**		
**Display 1**	**Frequent user**	
	Readings available on specific reader and mobile phones (ability to choose)	Same as minimum including option of data display on the device in addition to the reader/mobile phones
	**Sporadic user**	
	Readings available on specific reader and mobile phones (ability to choose)	Same as minimum including option of data display on the device in addition to the reader/mobile phones
**Display 2**	**Frequent user**	
	Ability to set different languages; ability to display basic pictograms/alert colour	Large display option; colour display; pictograms; different languages; screen reading for visually impaired
	**Sporadic user**	
	Ability to set different languages; ability to display basic pictograms/alert colour	Large display option; colour display; pictograms; different languages; screen reading for visually impaired.
**On device data recording/storage**	**Frequent user**	
	Measurements from the last 3 months; data information retained in device in the absence of power source (e.g., during battery exchange) or other events disrupting device function	Measurements recorded/stored longer than 3 months; data information retained in device in the absence of power source (e.g. during battery exchange) or other events disrupting device function
	**Sporadic user**	
	Measurements from the last 6 months; data information retained in device in the absence of power source (e.g. during battery exchange) or other events disrupting device function.	Measurements recorded/stored longer than 6 months; data information retained in device in the absence of power source (e.g. during battery exchange) or other events disrupting device function
**Alerts and user guidance**	**Frequent user**	
	When glucose is in hypoglycaemic range; alert user-guidance in the form of visual display, noise, vibration (option to choose); for devices measuring continuously alerts should be provided when detected (even if the user does not actively access the device/reading)	Same as minimum, including alarms whenever outside of user-defined target range (hypo/hyper); alarms for trends/severity (customizable); reading reminder; temperature warning; ketone check reminder; range indication (linked to colour/pictogram guidance); connectivity loss alert; alarms available on the device itself as well as on the reader/mobile phone (option to choose); note: some alarms/user guidance’s only apply to devices that measure continuously
	NOTES: Some alarms/user guidances only apply to devices that measure continuously	
	**Sporadic user**	
	When outside of the user-defined target range; reading reminder; charging reminder; alert user-guidance in the form of visual display, noise, vibration (option to choose)	Same as minimum, including temperature warning
**Activity log**	**Frequent user**	
	Ability to add symbols to log insulin injection, food intake and physical activity (time stamped)	Ability to add descriptive details to log insulin injections, food intake, physical activities and other self-defined categories (time stamped)
	**Sporadic user**	
	Ability to record recent events or reasons for testing (e.g. sickness)	Same as minimum
**Connectivity of device to reader or other equipment**	**Frequent user**	
	Bluetooth or other wireless data transfer options	Same as minimum, including option for cable connection, based on commonly available connectivity outlets
	**Sporadic user**	
	Bluetooth or other wireless data transfer options	Same as minimum, including option for cable connection, based on commonly available connectivity outlets
**Data management/ sharing**	**Frequent user**	
	Companion digital tools for the device should make all required data available to the end user; data sharing should be customizable by the end user with specified parties; for data exchange standards the application should support FHIR or JSON for connections to systems such as DHIS2, EHRs, national registries; all data sharing must be traceable and transparent to the user	Same as minimum
	**Sporadic user**	
	The manufacturer shall maintain and publish a list of Android mobile devices and iOS versions, including all devices, newer and older versions, that have been determined compatible with the device/app	Shall be compatible with most Android and iOS mobile devices that are readily available in LMICs
**Compatibility mobile devices**	**Frequent user**	
	The manufacturer shall maintain and publish a list of Android mobile devices and iOS versions, including low-priced devices and older versions, that have been determined compatible with the device/app	Shall be compatible with most Android and iOS mobile devices that are readily available in LMICs
	**Sporadic user**	
	The manufacturer shall maintain and publish a list of Android mobile devices and iOS versions, including all devices, newer and older versions, that have been determined compatible with the device/app	Shall be compatible with most Android and iOS mobile devices that are readily available in LMICs
**Device initialization–user handling**	**Frequent user**	
	Simple, on-device (or reader/mobile phone) guidance, self-paced setup by the user, with images wherever possible; including instructions for process step controls to avoid errors	Same as minimum
	**Sporadic user**	
	Simple, on-device (or reader/mobile) guidance, self-paced setup by the user, with images wherever possible; including instructions for process step controls to avoid errors	Same as minimum
**Device initialization–set-up**	**Frequent user**	
	Device should be ready for measurement and result display within 30 minutes for devices that require a warm-up time and are intended to stay in place for subsequent readings; ready for measurement and result display within 10 seconds for devices that are only applied to the body for each reading	Same as minimum except that devices intended to stay in place for subsequent readings should be ready for measurement and result display within 5 minuets
	**Sporadic user**	
	Ready for measurement and result display within 30 minutes for devices that require a warmup time and are intended to stay in place for subsequent readings; ready for measurement and result display within 10 seconds for devices that are only applied to the body for each reading	Same as minimum except that devices intended to stay in place for subsequent readings should be ready for measurement and result display within 5 minutes
**Calibration**	**Frequent user**	
	User calibration possible but not required	Same as minimum
	NOTES: The measurement concept of the device (e.g. optical) may require different calibration data configuration	
	**Sporadic user**	
	Factory calibrated (no user calibration needed)	Factory calibrated (no user calibration needed) but with optional user calibration
**Power source**	**Frequent user**	
	Device and any additional equipment should have minimum dependency on power; if batteries are used, the device power requirement should be minimum so batteries can last for many months and commonly available battery types should be used	Rechargeable device, using power from renewable sources (e.g. solar power) of all required pieces of equipment; for rechargeable items, the charge should last several days (e.g. 7–14)
	**Sporadic user**	
	Device and any additional equipment should have minimum dependency on power; if batteries are used, the device power requirement should be minimum so batteries can last for many months and commonly available battery types should be used	Rechargeable device, using power from renewable sources (e.g. solar power) of all required pieces of equipment; for rechargeable items, the charge should last several days (e.g. 7–14)
**Operating conditions (for all pieces of equipment)**	**Frequent user**	
	Temperature: 5°C– 45°C Water: under water for 60 minutes (unless devices can be removed temporarily) Humidity: 10–70% relative humidity Adhesives should be sweat resistant and hypoallergenic	Temperature: 0°C– 50°C Water: under water for 60 minutes Humidity: 10–90% relative humidity Adhesives should be sweat resistant and hypoallergenic; if over-patches are recommended, sufficient numbers need to be provided with the device/sensor; dust and UV resistant
	**Sporadic user**	
	Temperature: 5°C– 45°C Water resistant / splash proof Humidity: 10–70% relative humidity	Temperature: 0°C– 50°C Water resistant / splash proof Humidity: 10–90% relative humidity Dust and UV resistant
**Storage conditions (for all pieces of equipment)**	**Frequent user**	
	Validated temperature: 0°C– 65°C Relative humidity: 10–90%	Same as minimum and dust and UV resistant
	**Sporadic user**	
	Validated temperature: 0°C– 65°C Relative humidity: 10–90%	Same as minimum and dust and UV resistant
**Shelf life unopened (where applicable)**	**Frequent user**	
	24 months based on date of production. For multi-component systems (e.g. separate sensor and transmitter), the shelf life of the component with the shortest shelf life should be 24 months	36 months based on date of production. For multi-component systems (e.g. separate sensor and transmitter), the shelf life of the component with the shortest shelf life should be 36 months
	**Sporadic user**	
	24 months based on date of production. For multi-component systems (e.g. separate sensor and transmitter), the shelf life of the component with the shortest shelf life should be 24 months	36 months based on date of production. For multi-component systems (e.g. separate sensor and transmitter), the shelf life of the component with the shortest shelf life should be 36 months
**Environmental aspects**	**Frequent user**	
	Minimum amount of non-degradable materials, including for application of the device; minimum amount of single-use disposable items; ecological responsible production process (e.g. with respect to emissions, water usage); social responsible production	No single-use disposable items; use of bio-degradable materials; energy efficient
	**Sporadic user**	
	Minimum amount of non-degradable materials, including for application of the device; minimum amount of single-use disposable items; ecological responsible production process (e.g. with respect to emissions, water usage); social responsible production	No single-use disposable items; use of bio-degradable materials; energy efficient
**PERFORMANCE AND RESULTS**		
**Linear range**	**Frequent user**	
	Quantifiable range: 2.2 to ≥33.3 mmol/L (≤40 to ≥600 mg/dL) Limit of detection ≤1.11 mmol/L (≤20 mg/dL)	Same as minimum
	**Sporadic user**	
	Quantifiable range: 3.00 to 33.3 mmol/L (54 to 600 mg/dL) Limit of detection ≤1.11 mmol/L (≤20 mg/dL)	≤1.11 to ≥33.3 mmol/L (≤20 to ≥600 mg/dL)
**Accuracy**	**Frequent user**	
	In case of continuous measurements: FDA iCGM special controls In case of only intermittent measurements: FDA OTC guidance or ISO 15197	Same as minimum or better
	**Sporadic user**	
	No minimum requirements are defined due to the absence of applicable guidance/standards; devices with performance below the optimal req. might be acceptable if disease management needs are met, they do not introduce undue risks for users, devices are not used for insulin dosing	In case of only intermittent measurements: FDA OTC guidance or ISO 15197
**Interferences**	**Frequent user**	
	Inferences testing according to CLSI EP07 and EP37; declaration to user of substances with significant interference; for optical devices: all skin phototypes; hematocrit where applicable	Same as minimum
	**Sporadic user**	
	Inferences testing according to CLSI EP07 and EP37; declaration to user of substances with significant interference; for optical devices: all skin phototypes	Same as minimum
**Traceability**	**Frequent user**	
	• For devices that do not measure glucose continuously: Declaration of traceability chain according to ISO17511 without indication of measurement uncertainty or analytical error • For devices that do measure glucose continuously: A traceability chain should be established that accurately reflects the measurement technology and sample type	• For devices that do not measure glucose continuously: Declaration of traceability chain according to ISO17511 with indication of measurement uncertainty or analytical error • For devices that do measure glucose continuously: Same as minimum with indication of measurement uncertainty or analytical error
	**Sporadic user**	
	• For devices that do not measure glucose continuously: Declaration of traceability chain according to ISO17511 without indication of measurement uncertainty or analytical error • For devices that do measure glucose continuously: A traceability chain should be established that accurately reflects the measurement technology and sample type	• For devices that do not measure glucose continuously: Declaration of traceability chain according to ISO17511 with indication of measurement uncertainty or analytical error • For devices that do measure glucose continuously: Same as minimum with indication of measurement uncertainty or analytical error
**Precision**	**Frequent user**	
	Precision assessment based on CLSI EP05 (minimally invasive can be measured in vitro)	Same as minimum
	**Sporadic user**	
	Precision assessment based on CLSI EP05	Same as minimum
**Results format**	**Frequent user**	
	Quantitative across linear range, qualitative if below or above linear range	Quantitative across entire measurement range
	**Sporadic user**	
	Quantitative across linear range, qualitative if below or above linear range; indication of ranges-only possible where this is acceptable and enough to inform the user; minimum of 5 range categories should be defined and adjustable by the user (low, low/normal, normal, high/normal, high)	Quantitative across linear range, qualitative if below or above linear range
**Results unit**	**Frequent user**	
	Plasma-equivalent; possibility to change between mg/dL and mmol/L if needed and allowed by local regulations	Same as minimum
	**Sporadic user**	
	Plasma-equivalent; possibility to change between mg/dL and mmol/L if needed and allowed by local regulations	Quantitative across linear range, qualitative if below or above linear range
**PURCHASE CONSIDERATION**		
**Price per month to end payer (including purchase of all equipment and subscription fees where applicable)**	**Frequent user**	
	• Total cost per month (including amortization for one-off purchases over 12 months): US$ 20 • The cost for one-off purchases such as a reader or a measurement device without consumables shall be no more than: US$ 20, which means a cost of testing per day is no more than US$ 0.6 (+ 12 months amortization for one-off purchases)	• Total cost per month (including amortization for one-off purchases over 12 months): US$ 3.7 • The cost for one-off purchases such as a reader or a measurement device without consumables shall be no more than: US$ 12, which means cost of testing per day is no more than US$ 0.09 (+ 12 months amortization for one-off purchases)
	**Sporadic user**	
	• Total cost per month (including amortization for one-off purchases over 12 months): US$ 20 • The cost for one-off purchases such as a reader or a measurement device without consumables shall be no more than: US$ 20, which means a cost of testing per day is no more than US$ 0.6 (+ 12 months amortization for one-off purchases)	• Total cost per month (including amortization for one-off purchases over 12 months): US$ 3.7 • The cost for one-off purchases such as a reader or a measurement device without consumables shall be no more than: US$ 12, which means cost of testing per day is no more than US$ 0.09 (+ 12 months amortization for one-off purchases)
**Business model**	**Frequent user**	
	Certified quality management system for medical devices (e.g. ISO 13485); application of risk management to medical devices (e.g. ISO 14971); post-market surveillance system and customer support in place	Same as minimum
	NOTES: Calculate based on the guideline-recommended testing frequency for people on insulin	
	**Sporadic user**	
	In case of one-off purchase of the device, minimum fee to obtain readings, set alarms or access additional features	One off purchase of device with no additional fees or costs to obtain a reading, alarm or access any features
**SAFETY AND STANDARDS**		
**Quality management**	**Frequent user**	
	Certified quality management system for medical devices (e.g. ISO 13485); application of risk management to medical devices (e.g. ISO 14971); post-market surveillance system and customer support in place	Same as minimum
	**Sporadic user**	
	Certified quality management system for medical devices (e.g. ISO 13485); application of risk management to medical devices (e.g. ISO 14971); post-market surveillance system and customer support in place	Same as minimum
**Regulatory approvals**	**Frequent user**	
	Approval by at least one of the following stringent regulatory authorities: CE, US FDA, Health Canada, Australia TGA, Japan	Same as minimum, including WHO-PQ approval (if available)
	**Sporadic user**	
	Approval by at least one of the following stringent regulatory authorities: CE, US FDA, Health Canada, Australia TGA, Japan	Same as minimum, including WHO-PQ approval (if available)

CE, Conformité Européene; CSLI, Clinical and Laboratory Standards Institute; DHIS2, District Health Information Software 2; HER, electronic health records; FDA, United States Food and Drug Administration; FHIR, Fast Healthcare Interoperability Resources; iCGM, integrated continuous glucose monitoring system; ISO, International Organization for Standardization; JSON, JavaScript Object Notation; LMICs, low- and middle-income countries; OTC, over the counter; TGA, Therapeutic Goods Administration; UV, ultraviolet; WHO PQ, World Health Organization prequalification.

## Discussion

This TPP for a new non-invasive or minimally invasive glucose self-monitoring technology suitable for use in LMICs was developed using a robust multi-step process, similar to that used for previous TPPs [7–17]. A draft TPP developed by an expert group and refined according to qualitative research in LMICs was reviewed via online survey. Following the survey, one characteristic with <75% agreement for both use cases, seven with <90% agreement for the frequent use case and three with <90% agreement for the sporadic use case were further discussed by the expert group and refined as appropriate. The final TPP has 39 characteristics across the categories of scope, device, utility requirements, performance and results, purchasing considerations, and safety and standards. It incorporates minimal and optimal requirements for each characteristic for both the frequent and sporadic use cases. Previous studies have suggested that adherence to self-monitoring of glucose among people living with diabetes in LMICs is sub-optimal [19–21]. Developing appropriate tools that empower people to manage their condition through provision of non-invasive or minimally-invasive blood glucose monitoring devices that meet their requirements has potential to improve adherence to self-monitoring, possibly leading to improved glycaemic control and quality of life.

The TPP aims to encourage the development of devices for the management of diabetes in LMICs, to address the increasing burden of diabetes in low-resource countries. Additionally, it may be used to assess existing devices to determine how well they might meet needs in LMIC settings. To ensure that the TPP addressed the needs and requirements of the end user, the development process not only incorporated in-country qualitative research, but also included people living with diabetes and their caregivers in the expert group to guide the process. This aligns with the recently published WHO framework for engagement with people living with non-communicable diseases [22]. Ongoing engagement with end-users from LMICs during development of glucose self-monitoring technologies designed to align with this TPP will be essential to ensure that user feedback is incorporated and their needs continue to be addressed.

While this TPP will inform developers on the key characteristics of a device for use in LMICs, it is challenging to define a one-size-fits-all solution for glucose self-monitoring for people living with diabetes. Different users may have different perceptions, needs and preferences relating to glucose self-monitoring technologies, for example whether they are used intermittently or continuously. Infrastructure, healthcare access and cost, environmental conditions, and social and cultural aspects may vary considerably from one country to another. Nevertheless, this TPP will provide developers with important data on requirements of people living with diabetes in LMICs. It may also be pertinent for people living in poverty in HICs and people affected by crises and conflicts.

Limitations of this methodology include the potential for the survey design to encourage agreement as the quickest route to completion, and the possibility of bias. For example, the sequence in which the characteristics were presented may have led to disproportionate importance being placed on certain requirements. The TPP may also require periodic updating as new technologies emerge. The TPP is intended to be a `living document’, with requirements to be regularly reviewed and adapted to accommodate evolving needs and technologies. Agreement levels in the online survey were high, and following publication of the TPP for open consultation, no comments were received. The high agreement levels in the online survey may be testimony to the value of involving people living with diabetes, their caregivers and their healthcare providers in the qualitative study and development process, enabling potential issues to be identified and addressed early. However, it could also indicate less familiarity with TPPs among the NCD community. Historically, TPPs have been more commonly used in the infectious diseases field [7–12].

In summary, this TPP aims to provide guidance to manufacturers looking to develop new glucose self-monitoring technologies suitable for use in LMICs. It also supports decision-makers to select appropriate existing devices with respect to their suitability for use in LMICs. As there is an increasing interest in LMIC markets among manufacturers and increasing demand for new self-monitoring technologies among people living with diabetes in LMICs, this TPP may be of particular value in promoting improved access to self-monitoring in these settings.

## References

[1] International Diabetes Federation. IDF Diabetes Atlas: 10th Edition. Available from: https://diabetesatlas.org/atlas/tenth-edition/ [Accessed 10 October 2023].

[2] Collaboration NCDRF. Worldwide trends in diabetes since 1980: a pooled analysis of 751 population-based studies with 4.4 million participants. Lancet 2016;387:1513–30. doi: 10.1016/S0140-6736(16)00618-8 27061677 PMC5081106

[3] American Diabetes A. 6. Glycemic Targets: Standards of Medical Care in Diabetes-2021. Diabetes Care 2021;44:S73–S84. doi: 10.2337/dc21-S006 33298417

[4] Shang T, Zhang JY, Thomas A, Arnold MA, Vetter BN, Heinemann L, et al. Products for Monitoring Glucose Levels in the Human Body With Noninvasive Optical, Noninvasive Fluid Sampling, or Minimally Invasive Technologies. J Diabetes Sci Technol 2022;16:168–214. doi: 10.1177/19322968211007212 34120487 PMC8721558

[5] Vasan A, Friend J. Medical Devices for Low- and Middle-Income Countries: A Review and Directions for Development. J Med Device 2020;14:010803. doi: 10.1115/1.4045910 32328210 PMC7164506

[6] Piaggio D, Castaldo R, Cinelli M, Cinelli S, Maccaro A, Pecchia L. A framework for designing medical devices resilient to low-resource settings. Global Health 2021;17:64. doi: 10.1186/s12992-021-00718-z 34158072 PMC8220789

[7] Ferreyra C, Osborn J, Moussy F, Alirol E, Lahra M, Whiley D, et al. Developing target product profiles for Neisseria gonorrhoeae diagnostics in the context of antimicrobial resistance: An expert consensus. PLoS One 2020;15:e0237424. doi: 10.1371/journal.pone.0237424 32870912 PMC7462286

[8] Ivanova Reipold E, Easterbrook P, Trianni A, Panneer N, Krakower D, Ongarello S, et al. Optimising diagnosis of viraemic hepatitis C infection: the development of a target product profile. BMC Infect Dis 2017;17:707. doi: 10.1186/s12879-017-2770-5 29143620 PMC5688443

[9] Mather RG, Hopkins H, Parry CM, Dittrich S. Redefining typhoid diagnosis: what would an improved test need to look like? BMJ Glob Health 2019;4:e001831. doi: 10.1136/bmjgh-2019-001831 31749999 PMC6830052

[10] Dittrich S, Tadesse BT, Moussy F, Chua A, Zorzet A, Tangden T, et al. Target Product Profile for a Diagnostic Assay to Differentiate between Bacterial and Non-Bacterial Infections and Reduce Antimicrobial Overuse in Resource-Limited Settings: An Expert Consensus. PLoS One 2016;11:e0161721. doi: 10.1371/journal.pone.0161721 27559728 PMC4999186

[11] Denkinger CM, Dolinger D, Schito M, Wells W, Cobelens F, Pai M, et al. Target product profile of a molecular drug-susceptibility test for use in microscopy centers. J Infect Dis 2015;211 Suppl 2:S39–49. doi: 10.1093/infdis/jiu682 25765105 PMC4425821

[12] MacLean EL, Miotto P, Gonzalez Angulo L, Chiacchiaretta M, Walker TM, Casenghi M, et al. Updating the WHO target product profile for next-generation Mycobacterium tuberculosis drug susceptibility testing at peripheral centres. PLOS Glob Public Health 2023;3:e0001754. doi: 10.1371/journal.pgph.0001754 37000774 PMC10065236

[13] Vetter B, Beran D, Boulle P, Chua A, de la Tour R, Hattingh L, et al. Development of a target product profile for a point-of-care cardiometabolic device. BMC Cardiovasc Disord 2021;21:486. doi: 10.1186/s12872-021-02298-7 34627153 PMC8501932

[14] Pelle KG, Rambaud-Althaus C, D’Acremont V, Moran G, Sampath R, Katz Z, et al. Electronic clinical decision support algorithms incorporating point-of-care diagnostic tests in low-resource settings: a target product profile. BMJ Glob Health 2020;5:e002067. doi: 10.1136/bmjgh-2019-002067 32181003 PMC7050342

[15] Kadam R, White W, Banks N, Katz Z, Dittrich S, Kelly-Cirino C. Target Product Profile for a mobile app to read rapid diagnostic tests to strengthen infectious disease surveillance. PLoS One 2020;15:e0228311. doi: 10.1371/journal.pone.0228311 31995628 PMC6988927

[16] White W, Kadam R, Moussy F. Target product profile for readers of rapid diagnostic tests. Bull World Health Organ 2023;101:331–40. doi: 10.2471/BLT.23.289728 37131947 PMC10140691

[17] Tobin M, Ferreyra C, Piton J, Kelly-Cirino C, Katz Z, Kadam R. Development of a target product profile for a One Health antimicrobial resistance surveillance service. Oxford Open Digital Health 2022;1. doi: 10.1093/oodh/oqac001

[18] Safary E. User requirements for non-invasive and minimally invasive glucose self-monitoring devices in low- and middle-income countries: a qualitative study in Kyrgyzstan, Mali, Peru and Tanzania. BMJ 2023;Submitted, under review. Doi 10.1136/bmjopen-2023-076685 PMC10875487 38367964

[19] Wambui Charity K, Kumar AMV, Hinderaker SG, Chinnakali P, Pastakia SD, Kamano J. Do diabetes mellitus patients adhere to self-monitoring of blood glucose (SMBG) and is this associated with glycemic control? Experiences from a SMBG program in western Kenya. Diabetes Res Clin Pract 2016;112:37–43. doi: 10.1016/j.diabres.2015.11.006 26655019

[20] Mogre V, Abanga ZO, Tzelepis F, Johnson NA, Paul C. Adherence to and factors associated with self-care behaviours in type 2 diabetes patients in Ghana. BMC Endocr Disord 2017;17:20. doi: 10.1186/s12902-017-0169-3 28340613 PMC5366118

[21] Mogre V, Johnson NA, Tzelepis F, Shaw JE, Paul C. A systematic review of adherence to diabetes self-care behaviours: Evidence from low- and middle-income countries. J Adv Nurs 2019;75:3374–89. doi: 10.1111/jan.14190 31453637

[22] World Health Organization. WHO framework for meaningful engagement of people living with noncommunicable diseases, and mental health and neurological conditions. Available from: https://www.who.int/publications/i/item/9789240073074 [Accessed 24 October 2023].

